# Prolonged diet-induced obesity in mice modifies the inflammatory response and leads to worse outcome after stroke

**DOI:** 10.1186/s12974-015-0359-8

**Published:** 2015-08-04

**Authors:** Samaneh Maysami, Michael J. Haley, Natalia Gorenkova, Siddharth Krishnan, Barry W McColl, Catherine B Lawrence

**Affiliations:** Faculty of Life Sciences, The University of Manchester, Oxford Road, Manchester, M13 9PT UK; Faculty of Medical and Human Sciences, The University of Manchester, Oxford Road, Manchester, M13 9PT UK; The Roslin Institute and R(D)SVS, University of Edinburgh Easter Bush, Midlothian, EH25 9RG UK

**Keywords:** Diet-induced obesity, Cerebral ischaemia, Stroke, Chemokines, Neutrophils

## Abstract

**Background:**

Obesity increases the risk for ischaemic stroke and is associated with worse outcome clinically and experimentally. Most experimental studies have used genetic models of obesity. Here, a more clinically relevant model, diet-induced obesity, was used to study the impact of obesity over time on the outcome and inflammatory response after stroke.

**Methods:**

Male C57BL/6 mice were maintained on a high-fat (60 % fat) or control (12 % fat) diet for 2, 3, 4 and 6 months when experimental stroke was induced by transient occlusion of the middle cerebral artery (MCAo) for either 20 (6-month diet) or 30 min (2-, 3-, 4- and 6-month diet). Ischaemic damage, blood–brain barrier (BBB) integrity, neutrophil number and chemokine expression in the brain were assessed at 24 h. Plasma chemokine levels (at 4 and 24 h) and neutrophil number in the liver (at 24 h) were measured. Physiological parameters (body weight and blood glucose) were measured in naïve control- and high-fat-fed mice at all time points and blood pressure at 3 and 6 months. Blood cell counts were also assessed in naïve 6-month control- and high-fat-fed mice.

**Results:**

Mice fed a high-fat diet for 6 months had greater body weight, blood glucose and white and red blood cell count but no change in systolic blood pressure. After 4 and 6 months of high-fat feeding, and in the latter group with a 30-min (but not 20-min) occlusion of the MCA, obese mice had greater ischaemic brain damage. An increase in blood–brain barrier permeability, chemokine expression (CXCL-1 and CCL3), neutrophil number and microglia/macrophage cells was observed in the brains of 6-month high-fat-fed mice after 30-min MCAo. In response to stroke, chemokine (CXCL-1) expression in the plasma and liver was significantly different in obese mice (6-month high-fat fed), and a greater number of neutrophils were detected in the liver of control but not obese mice.

**Conclusions:**

The detrimental effects of diet-induced obesity on stroke were therefore dependent on the severity of obesity and length of ischaemic challenge. The altered inflammatory response in obese mice may play a key role in its negative impact on stroke.

## Background

Stroke is a major cause of death and disability and a key health and socioeconomic burden, yet there is no widely applicable treatment, despite many promising candidates being identified in preclinical studies. One reason for this lack of translation could be due to experimental studies in stroke failing to adequately consider the underlying status of a typical stroke patient, who usually present with diabetes, hypertension, atherosclerosis, metabolic syndrome or obesity [1, 2].

Obesity is a major health problem worldwide, and in the UK in 2010, over a quarter of adults were classified as obese. Obesity is associated with several risk factors for stroke incidence and outcome including metabolic syndrome, hypertension, diabetes and hypercholesterolaemia. However, obesity in its own right has now been identified as an independent risk factor for stroke and is often associated with worse clinical outcome [3]. Consistent with this, ischaemic damage, blood–brain barrier (BBB) breakdown and the incidence of haemorrhagic transformation are increased in obese rodents in response to experimental stroke [4–8]. Most of these studies have been performed in rodents that are genetically deficient in the appetite-regulating adipokine leptin (*ob/ob* mice) or have a defective leptin receptor (*db/db* mice and *fa/fa* Zucker rat). In addition to its effects on energy homeostasis, leptin regulates other important biological functions such as the immune system [9], and acute leptin administration is neuroprotective against ischaemic injury in mice [10]. However, the effects of obesity on stroke outcome in obese *ob/ob* mice are not reversed by leptin administration [8] and are therefore likely to be due to factors associated with adiposity and not leptin deficiency. Recent data have shown that a deleterious effect of obesity on acute brain injury is also observed in rat [11, 12], mouse [5, 13] or gerbil [14] models of diet-induced obesity. Since mutations leading to leptin deficiency have been identified only in a small subset of obese humans, diet-induced obesity has greater clinical relevance [15]. However, the impact of diet-induced obesity on stroke outcome in mice remains to be characterised fully, as it is not known exactly when during the development of obesity the negative effects become apparent.

Obesity is now considered an ‘inflammatory and prothrombotic condition’ that is associated with elevated systemic and vascular pro-inflammatory profiles [16, 17]. Stroke induces a rapid systemic and central inflammatory responses including expression of pro-inflammatory cytokines and chemokines and multiple inflammatory and cellular responses in the bone marrow, liver and spleen, which negatively impact on outcome [18]. As the inflammatory response after experimental stroke is altered in obese *ob/ob* mice [7] and in mice made obese with a diabetic diet [13], changes in the inflammatory response may contribute to the detrimental effect of obesity on stroke. However, less is known about how diet-induced obesity affects the inflammatory response to stroke.

The aim of this study therefore was to determine when during the development of diet-induced obesity in mice a negative effect on stroke outcome is observed and to compare this to changes in the central and peripheral inflammatory response.

## Methods

### Mice and diets

C57BL/6 male mice (8-week-old; Harlan UK Limited, UK) were randomly assigned a high-fat diet (60 % energy from fat, 35 % fat content by weight, 13 % saturated fatty acids, 58G9, Test Diets®, supplied by IPS Product Supplies Ltd, UK) or control diet (12 % energy from fat, 5 % fat content by weight, 0.78 % saturated fatty acids, 58G7) and housed in groups of 4–5. Separate groups of mice were then maintained on their respective diets for 2, 3, 4 or 6 months. Each duration of feeding diet (i.e. time on diet) was performed as a separate experiment, and randomisation to diet was therefore done independently for each experiment. All mice were given *ad libitum* access to their respective diets and water and were housed at a constant ambient temperature of 21 ± 2°C on a 12-h light, 12-h dark cycle (lights on at 0800 h). All experimental procedures using animals were conducted in accordance with the United Kingdom Animals (Scientific Procedures) Act, 1986 and approved by the Home Office and the local Animal Ethical Review Group, University of Manchester.

### Measurement of physiological and haematological parameters

Body weight was monitored in all mice. In tail vein blood samples, blood glucose was measured using a hand-held glucose monitor (Accu-Check Aviva, Roche, UK) and blood cell counts were analysed using a haematometer (Pentra ES 60, Horiba Ltd, UK). Blood pressure was measured in conscious mice using a tail cuff system (BP-2000, Visitech Systems, USA).

### Focal cerebral ischaemia

Focal ischaemia was induced by transient middle cerebral artery occlusion (MCAο). Briefly, under isoflurane anaesthesia (in a mixture of 30 % oxygen and 70 % nitrous oxide), the carotid arteries were exposed and a 6–0 silicon rubber-coated monofilament (Doccol, USA) with a 2-mm tip (210 μm diameter, coating length 405 mm) was introduced into the external carotid artery and advanced along the internal carotid artery until occluding the origin of the MCA. Cerebral blood flow was monitored in all mice by laser-Doppler (Moor Instruments, UK), and middle cerebral artery occlusion (MCAo) was confirmed by a drop in cerebral blood flow of at least 2 0% of baseline. If this drop in blood flow was not attained, animals were excluded from the analysis. After 20 or 30 min (see below), the filament was withdrawn to establish reperfusion and the wound sutured. During surgery, core body temperature was monitored using a rectal probe and maintained at 37 ± 0.5 °C, using a homeothermic blanket (Harvard Apparatus, Kent, UK) and all mice were kept anaesthetised throughout the whole surgical procedure. During recovery all mice were given saline (0.5 ml, subcutaneously). In some groups, tail vein blood was taken immediately before MCAo (time 0), and at 4 and 24 h after reperfusion, and plasma obtained after centrifugation (13,000 × g, 10 min) was stored at −80°C until further use.

In separate experiments mice fed a control or high-fat diet for 2 (*n* = 9/group), 3 (*n* = 7–8/group), and 4 (*n* = 9–12/group) months, the MCA was occluded for 30 min. In the 6-month group, MCAo was induced for either 20 (*n* = 5–8/group) or 30 (*n* = 5-6/group) min. In a separate study, MCAo was induced for 30 min in 6-month high-fat- or control-fed mice (*n* = 5–6/group), and sham-operated animals (*n* = 5–6/group) were also prepared where the filament was advanced along the internal carotid artery and was retracted immediately. For all groups of animals, mice within a given cage were randomly assigned to undergo either MCAo or sham surgery or no surgical intervention (naïve). In total, 10 % of mice died during or after the surgical procedure and 8 % were excluded due to lack of drop in cerebral blood flow as described above. A reduction in sample size for some groups is therefore shown in the results section.

### Tissue processing

Twenty-four hours after MCAo, mice were terminally anaesthetised with isoflurane and perfused transcardially with 0.9 % saline, and samples of the liver, spleen and lung were taken, frozen and stored at −80 °C until analysis. In a separate study, the brain was also taken after saline perfusion, and from the ipsilateral and contralateral hemisphere, the striatum and cortex were dissected and frozen. Following perfusion with saline, animals were then perfuse-fixed with 4 % paraformaldehyde (PFA; in 0.1 M phosphate buffer, PB). Brains were removed and post-fixed (in 4 % PFA), cryoprotected (30 % sucrose in 0.1 M PB) and frozen in isopentane on dry ice. Coronal brain sections (30 μm) were cut on a freezing sledge microtome (Bright 8000–001, Bright Instrument Co Ltd, UK). The liver was also removed and post-fixed (in 4 % PFA) before embedding in paraffin wax. Sections of liver were cut at 5 μm with a rotary microtome and mounted onto slides.

### Measurement of ischaemic damage

Brain sections were stained with either cresyl violet or haematoxylin and eosin (H&E). The volume of ischaemic damage was calculated as described previously [19]. Briefly, areas of damage on cresyl violet-stained sections were directly transcribed onto brain maps at eight anatomically defined coronal levels (bregma levels; 2.22, 1.54, 0.98, 0.14, −0.58, −1.22, −1.82 and −2.54 mm as defined by [20]) and are therefore corrected for oedema. The area of damage at each level was then measured using ImageJ (NIH, Bethesda, MD, USA) and the volume calculated. The volume of damage was expressed as the total amount of ischaemic damage, which was the sum of the damage in the striatum, cortex and hippocampus and thalamus combined (‘other’).

### Immunohistochemistry

Immunoperoxidase labelling for neutrophils was performed on PFA-fixed brain or paraffin-embedded liver sections mounted onto slides. For liver, sections were deparaffinised, rehydrated and antigen retrieval performed by incubation in heated citrate buffer (10 mM sodium citrate, 0.05 % Tween 20). All sections were initially incubated in 0.3 % H_2_O_2_ in 0.1 M PB for 10 min to quench endogenous peroxidase activity. Non-specific binding of antibodies was blocked for 1 h with 2 % normal serum (Vector Laboratories, UK) from species in which the secondary antibody was raised. Sections were incubated with a rabbit anti-neutrophil antibody (SJC for neutrophils, 1:1000, kindly provided by Drs. Daniel Anthony and Sandra Campbell, University of Oxford, UK) before being incubated in an anti-rabbit biotinylated (for brain: 1:500, Vector Labs) or HRP-labelled polymer (for liver: EnVision Plus, Dako) secondary antibody for 30 min–2 h at room temperature. For the brain, signal amplification was then performed by incubating sections in avidin-biotin complex (Vectastain ABC Elite, Vector Labs). For peroxidase visualisation, sections were incubated in 3, 3′-diaminobenzidine solution (DAB; for the liver: EnVision Plus, or for the brain: SigmaFast DAB with metal enhancer, Sigma-Aldrich, UK). All tissues stained for neutrophils were then counterstained with Papanicoloau haematoxylin and coverslipped. For visualisation of microglia/macrophages in the brain, PFA-fixed brain sections were treated as above but incubated in a rabbit anti-Iba1 antibody (1:1000, Wako Chemicals) overnight at 4 °C. For assessment of BBB disruption, primary antibody was omitted and a biotinylated anti-mouse IgG secondary antibody used (1:500, Vector Labs) before incubation in DAB (no metal enhancer).

SJC-positive neutrophils were counted in a defined area of the liver and results expressed as number of neutrophils/area. For the brain, the number of neutrophils per section (3–7 sections depending on the region analysed) was counted in the ipsilateral cortex (1.54 to −2.7 mm), striatum (1.54 to −1.22 mm) and hippocampus (−1.22 to −2.7 mm). The average number of cells per section was then calculated and the group mean determined for each brain region. For Iba1-positive cells, three separate images were taken in the striatum and cortex (for the ipsilateral and contralateral hemisphere). The area of Iba1 staining was assessed in each image using the threshold function of ImageJ (NIH, Bethesda, USA), with the threshold value kept constant and verified on all images so that only the area of positive Iba1 staining was measured. The area of Iba1 staining was expressed as percentage increase from the contralateral hemisphere. For BBB disruption, the intensity of IgG staining was analysed (using ImageJ) in the contralateral and ipsilateral cortex, striatum and hippocampus (as defined above) and results expressed as percentage increase from the contralateral hemisphere.

### Chemokine protein analysis

Saline-perfused liver, spleen, lung and brain samples were homogenised in buffer (50 mM Tris–HCl, 150 mM NaCl, 5 mM CaCl_2_ and 0.02 % NaN_3_) containing 1 % Triton-X 100 and a protease inhibitor cocktail (Set I; Calbiochem, Merck Chemicals Ltd). All homogenates were centrifuged at 10,000 × *g* for 30 min at 4 °C. The supernatant from the liver samples was further ultra-centrifuged at 100,000 × g for 1 h at 4 °C. All supernatants were then stored at −20 °C until analysis. CXCL-1 and CCL3 were assessed due to their chemoattractant properties for neutrophils. Mouse CXCL-1 (KC) was analysed by cytometric bead array (BD Biosciences, UK) and mouse CCL3 (MIP-1α) by ELISA (Duoset; R&D Systems, UK), according to the manufacturer’s instructions. Cytokine concentrations were determined by reference to the relevant standard curves. For liver, spleen, lung and brain supernatant, protein concentration was assessed by a bicinchonic protein assay (BCA; Pierce Biotechnology, USA) and results expressed as picogram per milligram protein for the liver, spleen and lung and the ratio of ipsilateral/contralateral for the brain. For the liver, spleen and lung, a separate group of 6-month high-fat- or control-fed mice (naïve) were treated as above but in the absence of any intervention (sham or MCAo).

### Data and statistical analyses

For all analyses, data are represented as mean ± standard error of the mean (SEM). Sample sizes were determined by power calculation (*α* = 0.05, *β* = 0.2) of our previous data at http://www.stattools.net/SSizAOV_Pgm.php. For all ex vivo analyses, the investigator was blinded to diet (control or high-fat) and treatment (e.g. length of occlusion of MCA, sham or naive).

For two groups, parametric data were analysed using Student’s *t*-test and for data with unequal variances, a Welch’s correction was applied. All other data was analysed using a two-way ANOVA with diet and treatment (sham or MCAo) or duration of MCAo as the fixed factors followed by a Bonferroni test for multiple comparisons. *P* < 0.05 was considered significant.

## Results

### Characterisation of the experimental model of diet-induced obesity

In separate groups of mice, body weight gain was significantly increased (*P* < 0.001 for all groups) after a high-fat diet at 2, 3, 4 and 6 months when compared to control-fed mice (Table 1). High-fat feeding also caused a significant increase (*P* < 0.05 and *P* < 0.01) in blood glucose at all time points. Blood pressure was measured in mice fed with their respective diets for 3 or 6 months, and there was no significant effect of high-fat feeding on systolic or diastolic blood pressure at both time points.

**Table 1:** Physiological response to a high-fat diet in mice

	2 month		3 month		4 month		6 month	
	Control	High-fat	Control	High-fat	Control	High-fat	Control	High-fat
Body weight gain (g)	5.2 ± 0.6	16.3 ± 1.3***	6.0 ± 2.0	23.0 ± 0.7***	7.6 ± 0.6	25.8 ± 0.9***	9.6 ± 1.4	28.4 ± 1.8***
Glucose (mmol/l)	7.1 ± 0.3	8.9 ± 0.4**	9.4 ± 0.5	11.0 ± 0.5*	8.4 ± 0.4	10.1 ± 0.5*	8.9 ± 0.7	10.7 ± 0.5*
Systolic blood pressure (mmHg)	-	-	105.8 ± 2.7	106.0 ± 1.7	-	-	106.1 ± 2.9	114.6 ± 6.7
Diastolic blood pressure (mmHg)	-	-	47.6 ± 1.6	51.2 ± 4.9	-	-	49.9 ± 4.0	52.7 ± 3.4

Separate groups of mice were maintained on a high-fat or control diet for 2, 3, 4 or 6 months when body weight gain (g, from week 0 on diet), blood glucose (mmol/l) and systolic and diastolic blood pressure (mmHg, in 3 and 6 month groups only) were assessed

Data are mean ± standard error of the mean (SEM), *n* = 5–12/group

*P < 0.05, **P < 0.01, ***P < 0.001 versus control-fed mice. Student’s *t*-test

In the blood of mice fed a high-fat diet for 6 months, there was an increase in the total number of white blood cells (WBC), which was accounted for by a significant increase (*P* < 0.05 and *P* < 0.01) in neutrophil and lymphocyte cell numbers when compared to control-fed mice (Table 2). Red blood cell (RBC) number and size significantly increased in high-fat-fed mice as indicated by an increase in RBC count, mean corpuscular volume (MCV) and red cell distribution width (RDW). Haemoglobin concentration and haematocrit (HCT) were also higher compared to control-fed mice (Table 2).

**Table 2:** The effect of a high-fat diet on haematological parameters

	Control	High-fat diet
Total WBC (× 10^3^/μl)	19.3 ± 0.9	27.6 ± 3.4*
Neutrophils (×10^3^/μl)	1.84 ± 0.09	3.59 ± 0.8**
Lymphocytes (× 10^3^/μl)	17.2 ± 0.9	23.7 ± 2.6*
Monocytes (× 10^3^/μl)	0.01 ± 0.004	0.04 ± 0.01
Eosinophils (× 10^3^/μl)	0.08 ± 0.02	0.06 ± 0.02
Basophils (× 10^3^/μl)	0.13 ± 0.03	0.15 ± 0.04
LIC (× 10^3^/μl)	0.05 ± 0.01	0.07 ± 0.01
RBC (× 10^6^/μl)	8.3 ± 0.4	11.5 ± 0.7***
HGB (g/dl)	12.0 ± 0.5	17.2 ± 0.9***
HCT (%)	39.8 ± 1.7	57.3 ± 3.3***
MCV (fL)	48.3 ± 0.8	49.6 ± 0.4**
RDW (%)	12.1 ± 0.3	12.8 ± 0.2*
Platelets (× 10^3^/μl)	1200 ± 114	1353 ± 85
MPV (fL)	5.4 ± 0.1	5.3 ± 0.05
PCT (%)	0.11 ± 0.01	0.12 ± 0.01

Mice were maintained on a high-fat or control diet for 6 months. Blood obtained from the tail vein was analysed using a Pentra ES 60 haematometer

*HCT* haematocrit *HGB* haemoglobin, *LIC* large immature cells, *MCV* mean corpuscular volume, *MPV* mean platelet volume, *PCT* plateletcrit, *RBC* red blood cells, *RDW* red cell distribution width, *WBC* white blood cells

Data are mean ± standard error of the mean (SEM), *n* = 10–12/group

*P < 0.05, **P < 0.01, ***P < 0.001 versus control-fed mice. Student’s *t*-test

### Severity of obesity and ischaemic challenge interact to modify outcome after experimental stroke

**Fig. 1:**
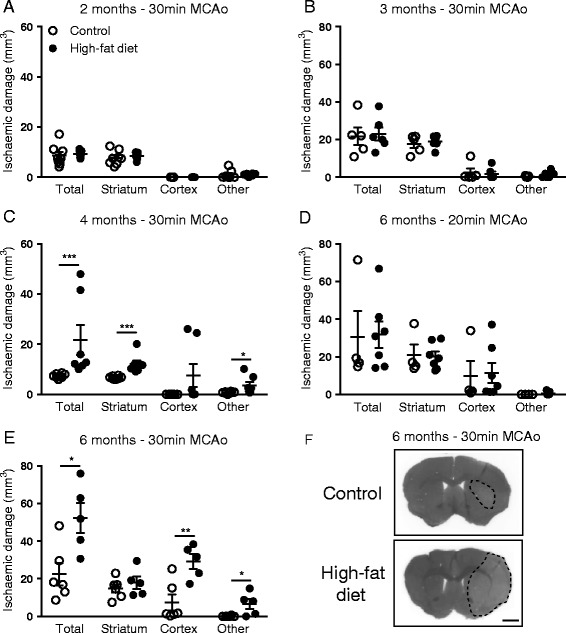
The detrimental effect of a high-fat diet on stroke outcome is dependent on the severity of obesity and length of ischaemic challenge. Male C57BL/6 mice (8-week-old) were maintained on either a control or high-fat diet and, in separate groups of mice, 30-min middle cerebral artery occlusion (MCAo) was performed after 2 (**a**), 3 (**b**), 4 (**c**) and 6 (**e**) months on diet. In mice fed diet for 6 months, MCAo was also performed for 20 min (**d**). Quantification of volume of ischaemic damage was performed on representative sections taken at eight defined coronal levels after 24-h reperfusion. **f** Representative pictures from 6-month high-fat- or control-fed mice after 30-min MCAo. Scale bar, 1.25 mm. Data are shown as mean ± standard error of the mean (SEM). *n* = 4–9/group. *P < 0.05, **P < 0.01, ***P < 0.001 versus control-fed mice for the same brain region. Student’s *t*-test

**Fig. 2:**
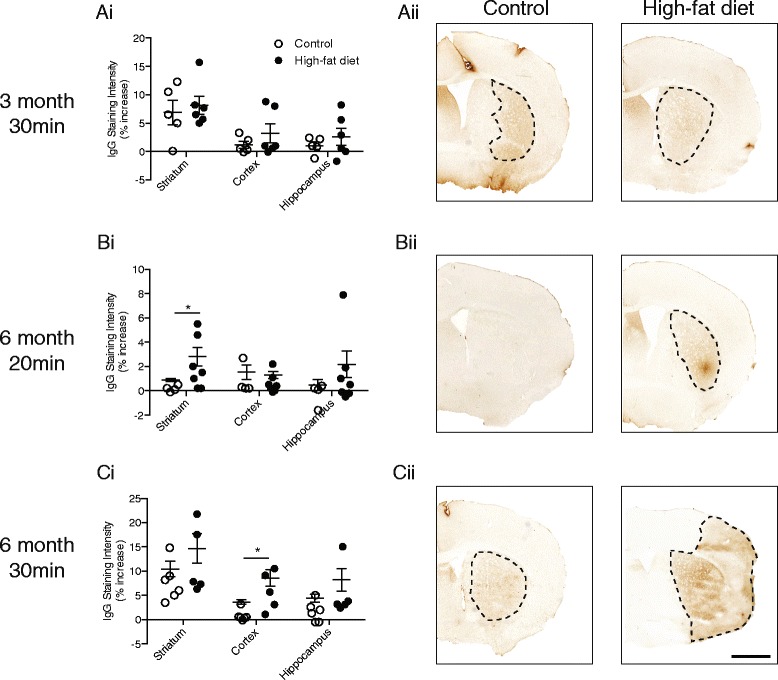
A high-fat diet disrupts the blood–brain barrier integrity after stroke. Male C57BL/6 mice (8-week-old) were maintained on either a control or high-fat diet and, in separate groups of mice, 30-min MCAo was performed after 3 (**ai**) and 6 (**ci**) months on diet. In mice fed diet for 6 months, MCAo was also performed for 20 min (**bi**). Blood–brain barrier permeability was assessed after 24-h reperfusion by measuring the staining intensity of immunoperoxidase-labelled IgG and is expressed as a percentage increase in intensity from the contralateral hemisphere. For this group of animals, the data for volume of ischaemic damage at 24 h is illustrated in Fig. 1b, d, e. **aii**–**cii** Representative sections of IgG immunohistochemistry on coronal brain sections. Scale bar, 1.25 mm. Data are shown as mean ± standard error of the mean (SEM). *n* = 4–7/group. *P < 0.05 versus control-fed mice for the same brain region. Student’s *t*-test

There was no significant effect of obesity on the volume of ischaemic damage after 30-min MCAo in mice fed a high-fat diet for 2 or 3 months (Fig. 1a, b). By 4 months of high-fat feeding, obese mice had an increase in the amount of total ischaemic damage in response to 30-min MCAo when compared to control-fed mice (*P* < 0.001 Fig. 1c), which was accounted for by an increase in damage in the striatum and ‘other’ brain areas. In obese mice fed a high-fat diet for 6 months, an exacerbation of total ischaemic damage was also observed after 30-min MCAo (*P* < 0.05 Fig. 1e, f) but not when the MCA was occluded for 20 min (Fig. 1d). This exacerbation of damage after 30-min MCAo in 6-month high-fat-fed mice was due to an increase in the cortex and ‘other’ brains regions rather than the striatum. Obesity did not appear to increase the risk of haemorrhagic transformation as no areas containing red blood cells were observed in obese (or control) mice at all time points or duration of ischaemic damage (when assessed by H&E staining). In the same group of animals, immunohistochemistry for IgG to assess the integrity of the BBB revealed that after 6 months of high-fat feeding, an increase in IgG staining intensity was observed in the cortex (*P* < 0.05) but not the striatum of obese mice in response to 30-min MCAo (Fig. 2ci–ii). After 20-min MCAo, there was a significant increase in the intensity of IgG staining in the striatum (*P* < 0.05) but not cortex in high-fat-fed mice (Fig. 2bi–ii). There was no significant difference in IgG staining in all brain regions analysed in mice fed a high-fat diet for 3 months compared to controls (Fig. 2ai–ii). These data suggests that the impact of obesity on acute ischaemic brain injury is dependent on an interaction between the duration/severity of obesity and the ischaemic challenge.

### Elevated central chemokine expression is associated with greater brain neutrophil infiltration in obese mice in response to stroke

**Fig. 3:**
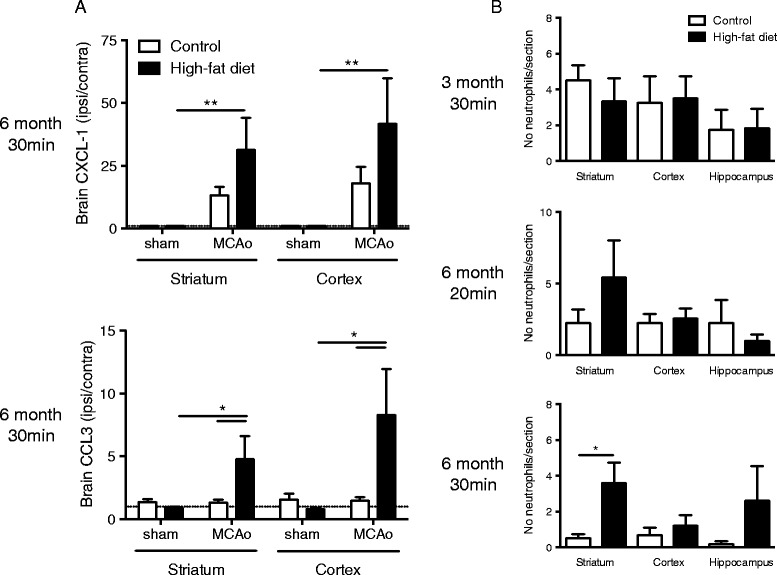
Diet-induced obesity increases chemokine expression and neutrophil number in the brain after stroke. Male C57BL/6 mice (8-week-old) were maintained on either a control or high-fat diet for 3 or 6 months. **a** In 6-month-fed mice, 30-min MCAo or sham surgery was performed. CXCL-1 and CCL3 protein expression were assessed in the striatum and cortex 24 h after reperfusion by CBA or ELISA, respectively. *Dashed lines* represent limit of detection. **b** In separate groups of mice, 30-min MCAo was performed after 3 and 6 months on diet. In mice fed diet for 6 months, MCAo was also performed for 20 min. The average number of neutrophils/section was assessed by immunohistochemistry after 24-h reperfusion. In (**b**) for this group of animals, the data for volume of ischaemic damage at 24 h is illustrated in Fig. 1b, d, e. Data are shown as mean ± standard error of the mean (SEM). For (**a**), *P < 0.05, **P < 0.01 versus sham group on the same diet, or versus control-fed mice for the same treatment; *n* = 5–6/group. Two-way ANOVA with Bonferroni’s. For (**b**), *P < 0.05 versus control-fed mice for the same brain region; *n* = 4–7/group. Student’s *t*-test

A significant increase in CXCL-1 and CCL3 was detected in the ipsilateral striatum and cortex of brains from 6-month high-fat-fed mice 24 h after 30-min MCAo when compared to high-fat-fed mice that underwent sham surgery (Fig. 3a). There was no significant difference in CXCL-1 and CCL3 expression in the brain of control mice 24 h after 30-min MCAo compared to the sham group. This chemokine response in 6-month high-fat-fed mice was associated with a significant increase in the number of neutrophils in the striatum 24 h after 30-min MCAo (Fig. 3b). No difference in neutrophil number was observed in the brains of 6-month high-fat-fed mice after 20-min MCAo or in 3-month high-fat-fed mice after 30-min MCAo (Fig. 3b). No neutrophils were detected in the contralateral (non-ischaemic) hemisphere in any group of control- or high-fat-fed mice (data not shown).

**Fig. 4:**
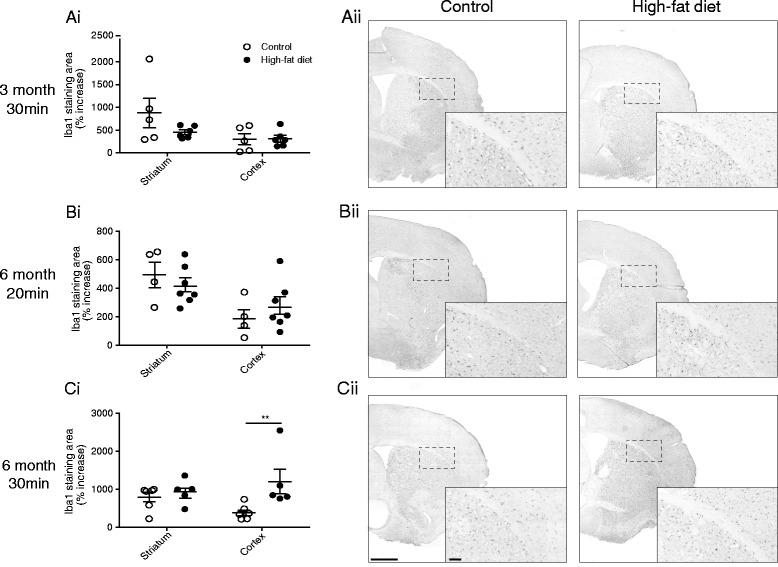
The effect of a high-fat diet on microglia/macrophages in the brain after stroke. Male C57BL/6 mice (8-week-old) were maintained on either a control or high-fat diet and, in separate groups of mice, 30-min MCAo was performed after 3 (**ai**) and 6 (**ci**) months on diet. In mice fed diet for 6 months, MCAo was also performed for 20 min (**bi**). After 24 h, microglia and macrophages were stained immunohistochemically using Iba1. The area of Iba1-positive cells was analysed and expressed as a percentage increase from the contralateral hemisphere. For this group of animals, the data for volume of ischaemic damage at 24 h is illustrated in Fig. 1b, d, e. **aii**-**cii** Representative coronal brain sections are shown for microglia/macrophage cells on coronal brain sections. Magnification of the area highlighted by the *dashed rectangle* is illustrated in the insert. Scale bar, 1.0 mm and 200 μm for the inserts. Data are shown as mean ± standard error of the mean (SEM). *n* = 4–7/group. **P < 0.01 versus control-fed mice for the same brain region. Student’s *t*-test

Immunohistochemistry to detect Iba1 showed an increase in the intensity of staining of microglia/macrophages in the area of infarct in sections from mice that had undergone MCAo compared to the contralateral non-ischaemic tissue. This increased staining was also associated with a change in morphology in some microglia cells. When quantified, there was no significant difference in the area of Iba1-positive cells between control- and high-fat-fed mice in the striatum and cortex at either 3 (after 30-min MCAo) or 6 (after 20-min MCAo) months of diet (Fig. 4). In 6-month high-fat-fed mice after 30-min MCAo, there was no difference in the area of Iba1-positive cells in the striatum but there was an increase in the cortex when compared to control-fed mice.

### Altered peripheral chemokine response is associated with an increase in liver neutrophils in control but not obese mice in response to stroke

**Fig. 5:**
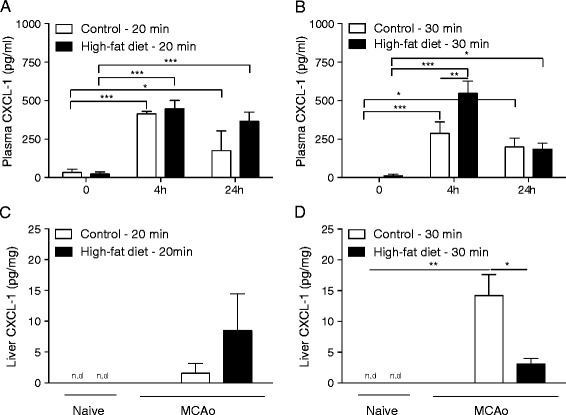
A high-fat diet alters the peripheral inflammatory response after stroke. Male C57BL/6 mice (8-week-old) were maintained on either a control or high-fat diet for 6 months and MCAo performed for either 20 or 30 min. CXCL-1 expression was analysed in the plasma (**a**, **b**) at 0, 4 and 24 h after reperfusion. In the liver (**c**, **d**) CXCL-1 expression was analysed at 24 h after MCAo and data compared to naïve mice. For this group of animals, the data for volume of ischaemic damage at 24 h is illustrated in Fig. 1d, e. n.d., not detected. Data are shown as mean ± standard error of the mean (SEM). *n* = 4–7/group. *P < 0.05, **P < 0.01, ***P < 0.001. Two-way ANOVA with Bonferroni’s

An increase in plasma CXCL-1 was observed 4 and 24 h after 20 or 30-min MCAo in mice fed a control or high-fat diet for 6 months compared to 0 hour (Fig. 5a, b). At 4 h after 30-min MCAo, the increase in plasma CXCL-1 was significantly greater in mice fed a high-fat diet for 6 months compared to control-fed mice (Fig. 5b). There was no significant change in liver CXCL-1 expression in either control or high-fat-fed mice 24 h after 20-min MCAo when compared to naïve mice (Fig. 5c). After 30-min MCAo, a significant increase in CXCL-1 was detected at 24 h in the liver of mice fed a control diet for 6 months, but no change was observed in high-fat-fed mice compared to naïve mice (Fig. 5d). This same pattern of expression of CXCL-1 in control and obese mice (at 6 months) was also seen in the spleen and lungs after 20 and 30-min MCAo (data not shown). There was no change in the expression of CCL3 in the plasma (4 and 24 h) or liver (at 24 h) in response to 20 or 30-min MCAo in control or 6-month high-fat-fed mice and all values were below the limit of detection (data not shown).

**Fig. 6:**
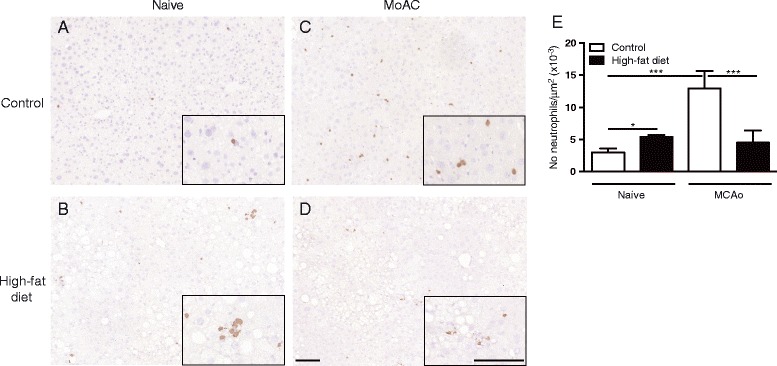
Stroke-induced neutrophil migration into the liver is impaired in mice fed a high-fat diet. Male C57BL/6 mice (8-week-old) were maintained on either a control or high-fat diet for 6 months when 30-min MCAo was performed. Representative photomicrographs illustrating immunohistochemical detection of neutrophils in the liver of naïve control (**a**) and high-fat fed (**b**) mice, and after MCAo in control (**c**) and high-fat-fed (**d**) mice. Scale bars, 100 μm. **e** The average number of neutrophils/area was assessed after 24-h reperfusion. For this group of animals, the data for volume of ischaemic damage at 24 h is illustrated in Fig. 1e. Data are shown as mean ± standard error of the mean (SEM). *n* = 5–6/group. *P < 0.05, ***P < 0.001. Two-way ANOVA with Bonferroni’s

In naïve mice fed a high-fat diet for 6 months, an increase in the number of neutrophils was observed in the liver, where neutrophils often appeared in clusters (Fig. 6a, b, e). In response to stroke (30-min MCAo), a significant increase in the number of neutrophils were detected at 24 h in the liver in control mice but there was no change in high-fat-fed mice when compared to naïve mice (Fig. 6a–e).

## Discussion

The present study demonstrates that obesity in mice due to a high-fat diet significantly alters the inflammatory response and increases the severity of ischaemic damage after stroke. Specifically, this study is the first to show that in mice, this effect of obesity on stroke outcome was dependent on both the severity of obesity and duration of ischaemic challenge. This detrimental effect of obesity in mice was observed after 4–6 months of high-fat feeding, suggesting that the mechanisms underlying the obesity-induced sensitivity to stroke take time to evolve. Furthermore, the duration of occlusion was also critical in determining whether obesity led to worse ischaemic damage, indicating that a threshold level of ischaemia is required before the effect of obesity is seen. In rats, 3-month (but not 1 month) exposure to a high-fat diet worsens outcome after endothelin-induced ischaemia [11]. Together, these data suggest that the negative impact of obesity on stroke outcome is critically dependent on how long the obese phenotype is present and the severity of the initial stroke insult.

Obesity is often associated with hypertension and insulin resistance/type II diabetes, complications that can also lead to a negative effect on stroke outcome clinically and in experimental models [21, 22]. As a high-fat diet up to 6 months did not cause hypertension, it is not likely that changes in blood pressure account for the negative effect of obesity seen here. Although, it is possible that blood pressure could change in obese mice in response to ischaemia, which was not measured here. A high-fat diet increased blood glucose in all mice. It is unlikely that hyperglycemia at the time of stroke is responsible for worse damage in obese mice as raised blood glucose was observed at all time points including at 2–3 months when obese mice did not have worse damage. Whether raised blood glucose for a prolonged period of time results in structural/physiological alterations that increase the susceptibility to an ischaemic insult remains to be tested. Furthermore, as insulin sensitivity and tolerance to glucose were not tested in the present study, it is possible that insulin resistance may have correlated with worse outcome.

Obesity in humans is associated with an increase in the number of blood cells that can lead to an increase in blood viscosity [23, 24]. Here, we show that obese mice (after 6-month high-fat diet) had higher levels of both RBC and WBC and an increase in haematocrit (percentage of RBCs in blood). These changes in blood cell number could potentially result in raised blood viscosity, although this remains to be determined here. Previous studies have shown that high-fat feeding in animals can significantly increase the number of WBC in specific lymphocyte subtypes and enhance blood viscosity [25–27]. As elevated haematocrit is associated with worse outcome in stroke patients [28, 29] and after experimental stroke in mice [30], these changes in blood cell number after high-fat feeding might contribute to worse outcome in obese mice.

In the present study, an increase in BBB permeability to IgG was observed in obese mice, which confirms that obesity promotes severe disruption to the BBB [8]. It is possible that the enhanced BBB disruption is due to the greater amount of ischaemic damage in obese mice; however, an increased BBB permeability was also seen in obese mice that had the same amount of damage in the striatum after 20-min MCAo compared to controls. These data indicate that obesity can lead to detrimental effects on cerebrovascular integrity after stroke independently of the extent of ischaemic damage and suggest multiple ways in which obesity may complicate stroke.

Obesity has also been shown to result in haemorrhagic transformation in response to cerebral ischaemia, an observation that was not seen here in diet-induced obese mice. However, the experimental studies in rodents reporting an increase in the incidence of haemorrhagic transformation have used different models of obesity including the leptin-deficient *ob/ob* mouse [8] and high-fat-fed rats [12] or a different model of cerebral ischaemia [5]. The reason why diet-induced obese mice are less susceptible to haemorrhagic transformation is unknown, but the enhanced ischaemic damage in these obese mice cannot be due solely to haemorrhagic transformation.

Stroke results in a local inflammatory response in the brain characterised by changes in cytokine and chemokine expression and immune cell infiltration [18] and activation of resident microglia cells [31]. There was no clear difference in the extent of activation of microglial (or macrophage) cells in the brain of obese mice apart from an increase in the cortex of 6-month high-fat-fed mice after 30-min MCAo. This change in microglial cells likely reflects the greater amount of ischaemic damage observed in this group of obese mice. Diet-induced obesity resulted in a greater number of neutrophils in the brain (striatum) of mice that had the worse outcome in response to stroke. Neutrophil infiltration was also reported to occur in adipose tissue after consumption of a high-fat diet in naïve rodents [32]. However, as obesity alone did not cause neutrophil invasion into the brain, these data suggest that neutrophil recruitment seen here occurs in response to ischaemia. The increase in brain chemokine expression (CXCL-1 and CCL3) and greater circulating blood neutrophil numbers that were observed in obese mice in the present study are likely responsible for the enhanced neutrophil infiltration into the brain in response to stroke. In support, chemokine expression and the number of neutrophils have been reported to be up-regulated after stroke in the brain of genetic models of obesity [4, 6]. As neutrophils have been shown to contribute to ischaemic brain damage [33, 34], it is possible that enhanced chemokine expression and neutrophil numbers in the brain of obese mice is responsible for the worse ischaemic damage in obesity. The increase in brain chemokine expression observed here is in contrast to that in mice fed a high-fat ‘diabetic’ diet where a blunted chemokine (CCL2) and cytokine (interleukin-6) response after stroke is observed [13]. These discrepancies highlight important differences in the inflammatory response to stroke in mice fed high-fat diets and are likely dependent on a number of factors including the type of diet, length on diet, duration of ischaemia and time after the ischaemic challenge.

A rapid and transient peripheral inflammatory response is also observed after stroke that precedes the local inflammatory response in the brain [35, 36]. In the present study, enhanced chemokine (CXCL-1) expression was observed in the blood at 4 h after stroke in obese mice that had greater damage. An increase in circulating neutrophils after experimental stroke has been observed by 8 h [34]. Although neutrophil numbers in the blood were not analysed here in response to stroke, it is possible that more neutrophils were mobilised from the bone marrow in response to early increase (4 h) in CXCL-1 expression in obese mice. An increase in the hepatic expression of CXCL-1 has also been reported after experimental stroke [35]. Here, we also observed an increase in hepatic expression of CXCL-1 expression in control (but not obese) mice, which was associated with neutrophil infiltration into the liver. This study is therefore the first to show that neutrophils migrate to the liver after experimental stroke in mice without co-morbidities, including obesity. Hepatic CXCL-1 and neutrophil numbers have also been shown to increase after an inflammatory stimulus in the brain [37]. The consequence of this inflammation in the liver after stroke is unclear but it might be involved in multi-organ dysfunction syndrome that can occur after cerebral ischaemia and is a significant source of morbidity and mortality in patients [38]. In support, liver damage has been reported to increase after experimental stroke in rats [39].

The inflammatory response in the liver of control mice after stroke was not observed in obese mice that had worse ischaemic brain damage and greater brain neutrophil migration. The lack of neutrophil trafficking into the liver of obese mice in response to stroke is consistent with the absence of CXCL-1 expression in this organ of these mice. A blunted inflammatory response in the liver of diet-induced obese mice also occurs after a peripheral inflammatory challenge [40] or infection [41]. Furthermore, in response to hepatic ischaemia-reperfusion injury, less CXCL-1 and neutrophil extravasation is observed in the liver of obese mice [42]. Overall, these data suggest that the central and peripheral chemokine response after experimental stroke is different in high-fat-fed mice compared to controls. Whether these differences in chemokine expression are responsible for the greater migration of neutrophils seen in the liver of control mice versus the higher number of neutrophils in the brain of high-fat-fed mice remains to be determined.

In summary, the data in the present study suggest that obesity may exert a greater negative effect on stroke outcome when the stroke insult is more severe and/or occurs when the obese phenotype has developed more strongly. In humans therefore, it is likely that the potential risk of obesity on stroke outcome are seen only after someone has been obese for a critical/minimum period of time, and that the effect of obesity could be reversible if someone has been obese for a only a short period of time. However, it remains to be determined if/how the altered neuro/systemic inflammatory response seen in obesity contributes to its detrimental effect on stroke outcome.

## Abbreviations

BCA: bicinchonic protein assay
BBB: blood–brain barrier
DAB: 3: 3′-diaminobenzidine solution
ELISA: enzyme-linked immunosorbent assay
HCT: haematocrit
HGB: haemoglobin
LIC: large immature cells
MCV: mean corpuscular volume
MCA: middle cerebral artery
MCAo: middle cerebral artery occlusion
MPV: mean platelet volume
PCT: plateletcrit
PFA: paraformaldehyde
PB: phosphate buffer
RBC: red blood cells
RDW: red cell distribution width
SEM: standard error of the mean
WBC: white blood cells
